# Effects of combined diet and physical activity on glycemic control and body composition in male recreational athletes with type 2 diabetes mellitus

**DOI:** 10.3389/fendo.2025.1525559

**Published:** 2025-06-06

**Authors:** Kayri Chouk, Raoua Triki, Ismail Dergaa, Halil İbrahim Ceylan, Houda Bougrine, Muntean Raul-Ioan, Abderraouf Ben Abderrahman

**Affiliations:** ^1^ High Institute of Sport and Physical Education of Ksar Said, University of Manouba, Manouba, Tunisia; ^2^ Physical Education of Sports Teaching Department, Faculty of Sports Sciences, Ataturk University, Erzurum, Türkiye; ^3^ Physical Activity Research Unit, Sport and Health (UR18JS01), National Observatory of Sports, Tunis, Tunisia; ^4^ High Institute of Sport and Physical Education Gafsa, Gafsa University, Gafsa, Tunisia; ^5^ Department of Physical Education and Sport, Faculty of Law and Social Sciences, University “1 Decembrie 1918” of Alba Iulia, Alba Iulia, Romania

**Keywords:** dietary intervention, diabetes mellitus, exercise therapy, glycemic control, insulin sensitivity, lipid metabolism, muscle composition, sports physiology

## Abstract

**Background:**

Type 2 diabetes mellitus (T2DM) is associated with metabolic and cardiovascular complications. While physical activity and dietary modifications are established interventions, their combined effects in active male populations remain underexplored.

**Objective:**

To assess the effects of a 12-week therapeutic diet and/or physical activity program on body composition and biochemical parameters in male recreational athletes with T2DM.

**Methods:**

Thirty-six male recreational athletes (aged 30–45 years) with T2DM were randomly assigned to: Physical Activity (PA), Nutritional Regimen (NR), Combined PA and NR (PA+NR), or Control (CG) groups. Body composition (body mass, body fat percentage, muscle mass) and biochemical markers (FBS, HbA1c, LDL-C, HDL-C, HOMA-IR) were measured at baseline, midpoint, and intervention end.

**Results:**

The PA+NR group showed significant improvements in body composition: reduced body mass (p<0.05, ES=0.29), reduced body fat percentage (p<0.05, ES=0.31), and increased muscle mass (p<0.05, ES=0.35). This group also demonstrated significant improvements in biochemical markers: reduced FBS (p<0.05, ES=0.17), HbA1c (p<0.05, ES=0.23), LDL-C (p<0.05, ES=0.17), and HOMA-IR (p<0.05, ES=0.39), with increased HDL-C (p<0.05, ES=0.29). The PA group showed only HbA1c reductions, while the NR group primarily improved lipid profiles. No significant changes occurred in the control group.

**Conclusion:**

A combined therapeutic diet and physical activity program effectively improved body composition and biochemical parameters in recreational athletes with T2DM. This combined approach could be recommended as an integral strategy for diabetes management in physically active individuals.

## 1 Introduction

Type 2 diabetes mellitus (T2DM) is a chronic metabolic disorder characterized by insulin resistance and progressive β-cell dysfunction, affecting over 463 million individuals worldwide and presenting significant health and economic challenges (1, 2). The development of T2DM is facilitated by several modifiable risk factors, including obesity, physical inactivity, poor dietary habits, and smoking, which collectively contribute to insulin resistance and metabolic dysfunction (1, 3). These factors precipitate the onset of T2DM but also exacerbate its progression, leading to life-threatening complications such as myocardial infarction, stroke, and premature mortality, emphasizing the critical importance of targeted lifestyle interventions in both prevention and management strategies (4).

While T2DM is commonly associated with sedentary lifestyles and obesity, it is important to recognize that it can also develop despite regular physical activity (5). Factors such as genetic predisposition, diet, metabolic dysfunction, and aging can contribute to the onset of T2DM (6). Athletes with T2DM face a unique challenge in maximizing performance while minimizing the disruption to optimal blood glucose management (5). They must carefully balance their training intensity, dietary intake, and medication use to prevent glycemic fluctuations that could impair performance or increase health risks (7). However, limited research has specifically explored the physiological adaptations and metabolic responses of athletes with T2DM following specific training and a diet program.

Physical activity (PA) is a cornerstone in T2DM management because it can enhance insulin sensitivity and promote glucose uptake in skeletal muscle, primarily through insulin-independent mechanisms. During PA, activation of AMP-activated protein kinase (AMPK) increases GLUT-4 translocation to muscle cell surfaces, facilitating glucose uptake independent of insulin, which is especially valuable in overcoming insulin resistance (8, 9). This effect makes PA an essential intervention for managing T2DM, helping to stabilize blood glucose levels and reduce glycemic excursions (10).

Among the different PA modalities, the combination of aerobic and resistance exercise is particularly effective in managing T2DM (11). Aerobic exercise enhances insulin sensitivity and lipid profile, increasing cardiovascular fitness by strengthening the heart muscle and facilitates glucose uptake by skeletal muscles contributing to better glycemic control, while resistance training increases muscle mass and strength, which plays a crucial role in glucose metabolism by enhancing insulin-mediated glucose disposal (12).

In addition to PA, dietary modifications are integral to T2DM management. A hypocaloric diet supports weight management and improves metabolic parameters by reducing systemic inflammation and enhancing insulin sensitivity (13). When paired with regular PA, dietary interventions have been shown to further optimize glycemic control, often improving both lipid profiles and blood pressure in individuals with T2DM (1, 14). While pharmacological treatments remain standard, lifestyle modifications, particularly the combination of PA and dietary changes, are increasingly recognized as sustainable and effective strategies for glycemic control and the reduction of cardiovascular risk in T2DM patients.

The integration of structured PA and a therapeutic diet may offer synergistic benefits in T2DM management, enhancing outcomes beyond what is typically achieved through either intervention alone. Recent studies indicate that combining PA with dietary modifications can result in substantial reductions in glycosylated hemoglobin (HbA1c) and improvements in lipid metabolism (15, 16). However, research is scarce on the effects of this combined intervention in recreational athletes with T2DM, who may exhibit unique physiological responses due to their engagement in regular PA.

Although PA and therapeutic diets are individually known to aid glycemic control and improve lipid profiles in individuals with T2DM, the combined effects of these interventions, particularly in athletic populations, remain underexplored. Recreational athletes with T2DM may experience unique physiological adaptations due to regular physical engagement, but few studies have specifically examined how structured PA alongside a therapeutic diet impacts both metabolic and body composition markers in this group. Additionally, while studies have shown improvements in body mass, blood glucose levels, and insulin sensitivity with PA and dietary interventions, there is limited evidence on the comprehensive effect of these combined strategies in altering body composition, glycemic control, lipid profiles, and insulin resistance in physically active individuals.

Thus, this study aimed to investigate the effects of a 12-week therapeutic diet and/or physical activity program in recreational athletes with T2DM, specifically focusing on (i) changes in body composition, including body mass, body fat percentage, and muscle mass; (ii) improvements in glycemic control and lipid profiles, including fasting blood sugar, HbA1c, low-density lipoprotein cholesterol (LDL-C), and high-density lipoprotein cholesterol (HDL-C); and (iii) reductions in insulin resistance, as measured by the Homeostasis Model Assessment of Insulin Resistance (HOMA-IR).

## 2 Materials and methods

### 2.1 Participants

Thirty-six male recreational athletes, aged 30 -45 years and engaged in regular structured sports activities with type 2 diabetes mellitus (T2DM), participated in this study. They were randomly assigned to one of four groups using a computer-generated random number: Physical Activity (PA, n=9), Nutritional Regimen (NR, n=9), Combined PA+NR (n=9), and Control (CG, n=9). Characteristics of participants are represented in **Table 1**. All participants engaged in individual sports, including wrestling and shot put. Inclusion criteria required that participants be non-smokers with no history of cardiovascular diseases and no use of medications other than metformin, which was provided by a physician (1). Participants also refrained from using ergogenic aids such as caffeine, alcohol, or recreational drugs during the experimental protocol. Written informed consent was obtained from each participant, and the study was conducted following the Declaration of Helsinki, with approval from the local University Ethics Committee on Human Research of the University of Manouba, Tunisia (Ethics Code: Tn, UM2019-73). It also complied with the ethical and procedural requirements for the conduct of sports medicine and exercise science research (17).

**Table 1:** Participants’ characteristics (means ± SD) (n=9 each group).

Variables		PA	NR	PA+NR	CG
Age (year)		40.7 ± 3.23	39.5 ± 2.89	41.5 ± 3.43	39.3 ± 3.19
Height (m)		1.80 ± 0.05	1.82 ± 0.15	1.76 ± 0.07	1.82 ± 0.09
Body mass (kg)		99.52 ± 5.68	97.95 ± 6.15	98.08 ± 6.65	98.35 ± 6.44
Time since diagnosis of T2DM (Year)		7.1 ± 2.76	6.5 ± 2.31	7.3 ± 3.07	7.1 ± 3.65
Type of Medication used		Metformin	Metformin	Metformin	Metformin
Sports engagement (Year)		5.2 ± 3.91	6.2 ± 4.12	6.3 ± 5.43	5.6 ± 4.12
Type of sport	Wrestler	25%	37.5%	50%	25%
	Shot putter	75%	62.5%	50%	75%

PA, Physical active group; NR, Nutritional regime group; PA+NR, physical active and nutritional regime group; CG, control group; T2DM, Type 2 Diabetes Mellitus.

### 2.2 Procedures

The sample size was determined using *a priori* power analysis with G*Power 3.1 (University of Kiel, Germany). Based on an F-test for repeated measures (within-between interaction), with an alpha level of 0.05, a power of 0.85, and an effect size of 0.23 derived from previous studies on the impact of exercise and diet in type 2 diabetes, a total of 36 participants was required to detect significant group differences (18). This calculation was cross-verified using ChatGPT-4, following sample size guidelines outlined by (19), and confirmed the same result of 36 participants.

### 2.3 Data collection

**Figure 1:**
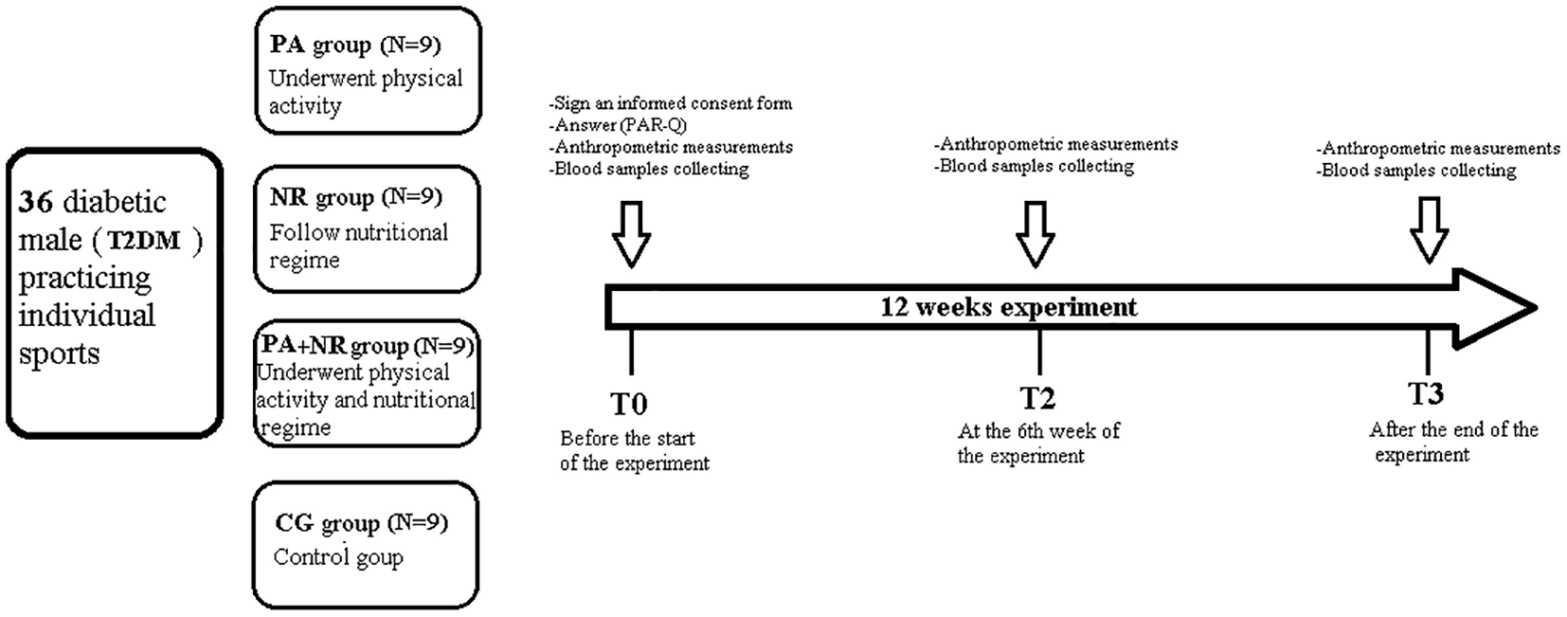
Overview of the Study Design. PA, Physical active group; NR, Nutritional regime group; PA+NR, Physical active and nutritional regime group; CG, control group; T0, Baseline measurements; T1, 6 weeks after the start of the intervention; T2, Measurements, twelve weeks after the start of the intervention; T2DM, Type 2 Diabetes Mellitus.

The study involved a 12-week intervention, preceded by a two-week familiarization period to standardize exercise techniques and dietary protocols. Measurements were taken before the start of the intervention (Baseline measurements) (T0), at the 6^th^ week of intervention (T1), and post- 12 weeks of intervention (T2). Participants were randomly assigned to one of four groups, each with specific intervention protocols: PA (physical activity only), NR (therapeutic diet only), PA+NR (combined physical activity and diet), and CG (no intervention). The intervention period ran from November 10, 2022, to January 15, 2023. The experimental design is represented in detail in **Figure 1**.

### 2.4 Training program

The PA intervention included a structured regimen of resistance and aerobic exercises, performed three times weekly on non-consecutive days (Saturday, Monday, and Wednesday) for a total of 60 to 65 minutes per session. Each session began with a 10-minute dynamic warm-up, including low-intensity running and stretching exercises. The resistance training component (25 minutes) targeted major muscle groups and progressed in intensity as follows: The total volume training was fixed at 45% of one-repetition maximum (1RM) (intensity) of 8 (repetitions)x 3 (sets) with 2-minute rest between sets and exercises during the 1^st^ week till the 4^th^ week of the experiment. The principle of progressive overload was applied to the periodized program, where the RT protocol started by performing 55% 1RM of 12 repetitions x3 sets during the weeks 5^th^ to 8^th^ weeks and progressed to 70% 1RM of 12 repetitions x 3 sets from the start of the 9^th^ till the finalization of the experiment (20). Exercises included leg presses, squats, lunges, push-ups, bench presses, and rows. Aerobic exercises (20 minutes) included treadmill jogging and cycling on an Orbitrac machine at 25-35% of heart rate reserve, followed by a 5–10-minute cool-down.

All training sessions were supervised by an experienced strength and conditioning practitioner to ensure correct technique and progressions were implemented appropriately.

### 2.5 Therapeutic nutrition program

Participants in the NR and PA+NR groups followed a hypocaloric diet to support gradual weight reduction. The Harris-Benedict equation was used to determine each participant’s basal metabolic rate, adjusted for activity level (21). Caloric intake began at 1500 kcal/day for the first three weeks, was reduced to 1200 kcal for weeks 4-6, and reached 800 kcal by week 9 with a frequency of 3 meals per day (breakfast, lunch, and dinner). The diet emphasized balanced macronutrients, approximately 50% carbohydrates, 20% proteins, and 30% fats, focusing on nutrient-dense, unprocessed foods. Participants were selected from pre-approved meal options using food-composition tables from the Tunisian National Institute of Statistics, designed to maintain glycemic stability and essential nutrient intake (22).

Dinner was recommended before 9 p.m., with no additional food intake outside the planned meals. Regarding energy deficits, participants’ caloric intake was carefully monitored, and while energy expenditure was not directly measured, the diet was adjusted based on individual activity levels and body composition goals. To ensure that micronutrient intake remained sufficient, special attention was given to meeting daily requirements for vitamins and minerals, such as vitamin D, calcium, and magnesium, which are critical for athletes with T2DM.

### 2.6 Anthropometric measurements

Body composition was assessed at T0, T1, and T2 using bioelectrical impedance analysis (BIA) (InBody 770, Germany), with daily calibration. A BIA was chosen due to its practicality, accessibility, and non-invasive nature, making it a feasible option for field-based studies with active participants. Measurements were conducted in a fasting state after an overnight fast and at least 36 hours post-exercise. Parameters included body mass (kg), body fat percentage (%), and muscle mass (kg). Participants stood on designated foot and hand placement areas on the device, with data on ID, age, gender, and height entered before measurements.

### 2.7 Blood tests

Fasting blood samples (15 ml) were collected from each participant between 8:00 and 10:00 a.m. after a minimum 12-hour fast and 48 hours without high-intensity exercise. Biochemical markers included Fasting blood sugar (FBS), glycated hemoglobin (HbA1c), total cholesterol (TC), high-density lipoprotein cholesterol (HDL-C), low-density lipoprotein cholesterol (LDL-C), triglycerides (TAG), and insulin resistance, assessed via the Homeostasis Model Assessment of Insulin Resistance (HOMA-IR). HbA1c and lipid profiles were analyzed using enzyme-linked assays, while HOMA-IR was calculated using the formula: fasting plasma insulin (µU/ml) x fasting plasma glucose (mmol/L)/22.5 (23). All blood samples were collected indoors and at the same time of day (between 9:00 and 10:00 a.m.) to minimize the effects of diurnal variations in the measured biochemical variables.

### 2.8 Statistical analysis

Data analysis was performed using SPSS version 21.0 (IBM Corp, Armonk, NY). The Kolmogorov-Smirnov test assessed data normality. A two-way ANOVA for repeated measures (time x group) was used to compare parameters across T0, T1, and T2, with Bonferroni adjustments for *post hoc* tests to identify significant within-group and between-group differences over time. Effect sizes (ES) were calculated and classified using Cohen’s d, with thresholds of trivial (<0.2), small (0.2-0.6), moderate (0.6-1.2), large (1.2-2.0), and very large (>2.0) according to Hopkins’ criteria. Statistical significance was set at p < 0.05 (24).

## 3 Results

All participants completed the study without injuries or reported adverse effects. **Table 2** provides data on body composition for all groups across T0, T1, and T2.

**Table 2:** Changes in body composition (mean ± SD) at T0, T1, and T2 among all groups (n=9 for each group).

Variables	Group	T0	T1	T2	Group effect (ES)	Time effect (ES)	Group x Time effect (ES)
Body Mass Index (Kg/m^2^)	PA	99.52 ± 5.68	96.76 ± 5.36*	95.75 ± 5.18*	0.001 (0.18)	0.3 (0.26)	0.01 (0.29)
	NR	97.95 ± 6.15	95.78 ± 6.16*	94.47 ± 5.24*			
	PA+NR	98.08 ± 6.65	92.68 ± 5.06**	83.40 ± 6.10**			
	CG	98.35 ± 6.44	99.87 ± 7.78	97.56 ± 7.34			
Body Fat (%)	PA	32.56 ± 4.13	29.78 ± 3.63*	27.21 ± 2.38*	0.02 (0.23)	0.04 (0.35)	0.03 (0.11)
	NR	32.172 ± 3.49	27.87 ± 3.59*	24.51 ± 1.35**			
	PA+NR	31.82 ± 3.61	28.70 ± 3.39*	24.71 ± 1.88*			
	CG	31.97 ± 3.41	32.12 ± 3.33	31.74 ± 1.64			
Muscle Mass (Kg)	PA	37.18 ± 4.26	39.43 ± 3.29*	42.01 ± 4.16*	0.01 (0.17)	0.04 (0.21)	0.001 (0.35)
	NR	37.17 ± 5.43	37.26 ± 5.42	37.53 ± 4.45			
	PA+NR	36.24 ± 5.06	38.20 ± 5.14*	41.03 ± 5.23*			
	CG	36.81 ± 5.67	37.08 ± 5.26	36.94 ± 5.14			

PA, Physical active group; NR, Nutritional regime group; PA+NR, physical active and nutritional regime group; CG, control group; T0, Baseline measurements; T1, 6 weeks after the start of the intervention; T2, measurements, twelve weeks after the start of the intervention, ES, Effect size; SD, Standard deviation; *p< 0.05 (in comparison with T0); **p< 0.01 (in comparison with T0).

### 3.1 Body composition measurements

A significant group-by-time interaction effect was observed across groups for body mass (p=0.01; ES=0.29), body fat percentage (p=0.03; ES=0.11), and muscle mass (p=0.001; ES=0.35). *Post hoc* analysis showed statistically significant reductions in body mass at T1 and T2 for the PA group (T1: p=0.02, ES=0.32; T2: p=0.03, ES=0.27), the NR group (T1: p=0.04, ES=0.19; T2: p=0.01, ES=0.10), and the PA+NR group (T1: p=0.002, ES=0.12; T2: p=0.009, ES=0.18), compared to baseline.

Body fat percentage significantly decreased in the PA group at T1 (p=0.03, ES=0.29) and T2 (p=0.05, ES=0.31), the NR group at T1 (p=0.05, ES=0.38) and T2 (p=0.005, ES=0.21), and the PA+NR group at T1 (p=0.02, ES=0.21) and T2 (p=0.03, ES=0.19), compared to T0. Muscle mass increased significantly in the PA group at T1 (p=0.04, ES=0.25) and T2 (p=0.01, ES=0.31) and in the PA+NR group at T1 (p=0.03, ES=0.17) and T2 (p=0.01, ES=0.13) relative to baseline (**Table 2**).

### 3.2 Biochemical parameters

Biochemical data across groups at T0, T1, and T2 are summarized in **Table 3**. Significant group-by-time interactions were noted for FBS (p=0.01; ES=0.17), HbA1c (p=0.02; ES=0.23), LDL-C (p=0.04; ES=0.17), HDL-C (p=0.04; ES=0.29), and HOMA-IR (p=0.05; ES=0.39).

**Table 3:** Blood and biochemical changes (mean ± SD) at T0, T1, and T2 among all groups (n=9 each group).

Variables	Group	T0	T1	T2	Group effect (ES)	Time effect (ES)	Group x Time effect (ES)
**FBS** (**Mg/dl)**	**PA**	135.23 ± 9.81	134.91 ± 8.45	134.17 ± 10.09	**0.05** **(0.20)**	**0.04** **(0.34)**	**0.01** **(0.17)**
	**NR**	136.60 ± 11.59	135.10 ± 9.76	136.00 ± 9.42			
	**PA+NR**	138.50 ± 13.34	129.00 ± 8.09*	112.00 ± 10.59**			
	**CG**	138.98 ± 9.31	137.82 ± 7.39	137.07 ± 8.23			
**HbA1C (%)**	**PA**	8.43 ± 0.71	7.71 ± 0.32*	7.03 ± 0.31*	**0.03** **(0.35)**	**0.05** **(0.28)**	**0.02** **(0.23)**
	**NR**	8.67 ± 0.73	8.41 ± 0.69	8.42 ± 0.23			
	**PA+NR**	8.36 ± 0.86	7.29 ± 0.42*	6.68 ± 0.44*			
	**CG**	8.81 ± 0.56	8.99 ± 0.41	8.75 ± 0.58			
**TC (mg/dL)**	**PA**	241.04 ± 15.06	232.54 ± 14.45	221.57 ± 10.51	**0.06** **(0.48)**	**0.05** **(0.31)**	**0.7** **(0.37)**
	**NR**	237.32 ± 23.17	231.15 ± 10.95	220.00 ± 8.31			
	**PA+NR**	238.00 ± 21.49	221.00 ± 13.90	202.00 ± 7.52			
	**CG**	239.10 ± 18.59	241.07 ± 11.65	235.87 ± 10.16			
**TAG (mg/dL)**	**PA**	219.50 ± 22.51	210.50 ± 13.51	191.40 ± 7.87	**0.1** **(0.53)**	**0.06** **(0.29)**	**0.09** **(0.42)**
	**NR**	217.62 ± 18.38	208.48 ± 14.42	198.51 ± 11.10			
	**PA+NR**	217.50 ± 21.50	202.50 ± 15.36	182.50 ± 7.16			
	**CG**	215.50 ± 19.32	212.50 ± 11.36	214.50 ± 7.12			
**LDL-C (mg/dL)**	**PA**	158.51 ± 13.32	151.94 ± 9.41	148.56 ± 7.63	**0.03** **(0.22)**	**0.05** **(0.15)**	**0.04** **(0.17)**
	**NR**	162.94 ± 12.15	155.52 ± 7.32*	131.50 ± 7.87*			
	**PA+NR**	160.80 ± 12.28	143.90 ± 5.25**	131.50 ± 6.25**			
	**CG**	160.22 ± 11.48	162.65 ± 9.85	160.20 ± 8.11			
**HDL-C (mg/dL)**	**PA**	37.54 ± 6.13	38.42 ± 4.98	39.21 ± 8.28	**0.04** **(0.31)**	**0.03** **(0.37)**	**0.04** **(0.29)**
	**NR**	37.65 ± 5.13	47.27 ± 5.29*	41.43 ± 5.21*			
	**PA+NR**	36.80 ± 4.87	44.60 ± 4.50**	54.20 ± 8.28**			
	**CG**	36.54 ± 5.24	37.61 ± 4.32	37.73 ± 8.15			
**HOMA-IR**	**PA**	6.12 ± 2.33	5.27 ± 2.47	5.09 ± 2.00	**0.04** **(0.41)**	**0.06** **(0.41)**	**0.05** **(0.39)** **(0**
	**NR**	6.75 ± 2.52	5.97 ± 2.34	5.20 ± 1.77			
	**PA+NR**	6.70 ± 2.84	4.81 ± 2.07*	3.20 ± 0.67*			
	**CG**	6.81 ± 3.14	6.05 ± 2.87	5.65 ± 2.51			

PA, Physical active group; NR, Nutritional regime group; PA+NR, physical active and nutritional regime group; CG, control group; T0, Baseline measurements; T1, 6 weeks after the start of the intervention; T2, measurements, twelve weeks after the start of the intervention; ES, Effect size; SD, Standard deviation; FBS, Fasting blood sugar; HbA1C, Hemoglobin A1C; TC, Total cholesterol; TAG, Triglycerides; LDL-C, Low-density lipoprotein cholesterol; HDL-C, High-density lipoprotein cholesterol; HOMA-IR, Homeostatic model assessment for insulin resistance; *p< 0.05 (in comparison with T0); **p< 0.01 (in comparison with T0).

The PA+NR group showed significant reductions in FBS at T1 (p=0.02, ES=0.29) and T2 (p=0.001, ES=0.12), HbA1c at T1 (p=0.03, ES=0.31) and T2 (p=0.02, ES=0.20), and LDL-C at T1 (p=0.004, ES=0.12) and T2 (p=0.007, ES=0.15), as well as significant increases in HDL-C at T1 (p=0.003, ES=0.22) and T2 (p=0.002, ES=0.31). HOMA-IR significantly decreased at T1 (p=0.04, ES=0.41) and T2 (p=0.05, ES=0.42) for the PA+NR group.

The PA group displayed a significant reduction in HbA1c at T1 (p=0.02, ES=0.25) and T2 (p=0.03, ES=0.36) from baseline, while the NR group exhibited significant decreases in LDL-C at T2 (p=0.02, ES=0.24) and T2 (p=0.03, ES=0.36) and increases in HDL-C at T1 (p=0.04, ES=0.29) and T2 (p=0.04, ES=0.28), compared to T0 (**Table 3**).

## 4 Discussion

This study aimed to investigate the effects of a 12-week physical activity and/or nutritional regimen on recreational athletes with T2DM, focusing on body composition, glycemic control, lipid profiles, and insulin resistance. The main findings reveal that the combined intervention of physical activity and dietary management (PA+NR group) produced significant improvements across all measured parameters, including reductions in body fat, FBS, HbA1c, and LDL-C levels, along with increases in HDL-C. The PA group showed a positive effect on HbA1c, while the NR group primarily affected LDL-C and HDL-C levels.

### 4.1 Effects on body composition

The PA+NR group demonstrated significant decreases in body mass and body fat percentage, along with an increase in muscle mass. The PA group also showed improvements in body fat and muscle mass, although these changes were less pronounced than those observed in the PA+NR group, while the NR group showed reductions in body mass and body fat without a notable increase in muscle mass.

The improvements in body composition with the combined intervention are consistent with findings from previous studies, highlighting the synergistic effects of exercise and dietary modification on fat loss and muscle preservation in T2DM populations. (25) similarly reported that combined aerobic and resistance training, coupled with dietary adjustments, was highly effective in reducing body fat and maintaining muscle mass in diabetic patients. This dual approach supports greater overall energy expenditure, leading to more effective weight management, which is critical for individuals with T2DM who are managing insulin sensitivity and metabolic regulation.

The increase in muscle mass in the PA and PA+NR groups can be attributed to resistance training, which not only promotes hypertrophy but also improves insulin sensitivity. This aligns with (26), who found that resistance exercise elevates GLUT-4 protein expression in skeletal muscle, enhancing glucose uptake. From a practical perspective, incorporating resistance training into T2DM management is essential for maintaining muscle mass, which provides a larger storage site for glucose and helps prevent hyperglycemic episodes, reducing the burden on pancreatic insulin production. These findings support the recommendation that T2DM management protocols include structured resistance training to maximize body composition benefits and aid long-term glycemic control. Notably, the body composition changes observed in our athletic population differ somewhat from those typically reported in non-athletic T2DM patients. While research by Sigal et al. (16) suggests that sedentary individuals with T2DM typically experience modest reductions in body fat with combined interventions, our athletic participants showed more substantial improvements, particularly in the PA+NR group, with a reduction from 31.82% to 24.71% in body fat percentage. This enhanced response may be attributed to recreational athletes’ existing muscle mass and metabolic adaptations from prior training. Unlike non-athletes who often experience significant muscle loss during dietary interventions, as reported by the Look AHEAD Research Group (27), our athletic participants maintained better muscle mass preservation even during caloric restriction, suggesting a protective effect of prior athletic conditioning against diet-induced muscle catabolism.

### 4.2 Effects on glycemic control

The PA+NR group showed significant reductions in FBS and HbA1c levels, while the PA group achieved a modest improvement in HbA1c. This suggests that dietary control plays a critical role in stabilizing FBS, as it directly influences postprandial glucose fluctuations. (28) found similar outcomes, where combined exercise and dietary interventions reduced HbA1c levels by an average of 0.5-0.7%, reinforcing the efficacy of a combined approach.

The mechanisms behind these improvements in glycemic control are multifaceted. Physical activity increases insulin sensitivity, which enhances glucose uptake by skeletal muscles, while dietary modifications help control carbohydrate intake and prevent spikes in FBS. A previous study conducted by Ivy (29) illustrates that exercise stimulates GLUT-4 translocation to the muscle cell membrane, facilitating insulin-independent glucose uptake. This effect is extended post-exercise, contributing to sustained glucose regulation throughout the day. Given these insights, practical recommendations for T2DM patients should prioritize meal timing around physical activity sessions to capitalize on glucose uptake by active muscle tissues and reduce postprandial glucose peaks. The glycemic response in our athletic population showed some distinct patterns compared to non-athletic individuals with T2DM. In their comprehensive review, Colberg et al. (30) noted that combined exercise and dietary interventions in sedentary T2DM patients typically produce modest improvements in HbA1c levels, whereas our athletic participants in the PA+NR group demonstrated more substantial reductions (from 8.36% to 6.68%), suggesting potentially enhanced sensitivity to lifestyle modifications in athletic populations. This difference may be explained by recreational athletes’ greater muscle glycogen turnover and enhanced insulin signaling pathways developed through consistent training. Interestingly, while Boulé et al. (13) reported that exercise alone can improve glycemic control in previously sedentary individuals, our PA-only group showed minimal changes in FBS, suggesting that recreational athletes might require more intensive dietary control to achieve further improvements in glycemic parameters beyond those already established through their regular physical activity.

### 4.3 Effects on lipid profile

Significant improvements in lipid profiles, especially reductions in LDL-C and increases in HDL-C, were observed in the PA+NR and NR groups. In contrast, the PA group did not exhibit notable changes in lipid profiles, which underscores the role of dietary intervention in lipid metabolism. This finding aligns with a meta-analysis by Kodama et al. (31), which highlighted the impact of dietary changes, combined with physical activity, on improving lipid parameters, especially LDL-C and HDL-C.

Dietary modifications are particularly influential in lipid regulation due to the direct effects of dietary fats on cholesterol levels. Intake of saturated fats is associated with elevated LDL-C, whereas unsaturated fats support HDL-C elevation. Therefore, it is essential to include dietary education in T2DM management protocols, emphasizing the reduction of saturated fats and the inclusion of unsaturated fats to mitigate cardiovascular risks associated with dyslipidemia. A previous study that supported this approach noted that dietary changes independently contribute to lipid improvements and are critical for T2DM patients. Thus, while exercise has numerous benefits, diet remains the primary driver of favorable lipid changes, especially for patients at high cardiovascular risk (32). When comparing lipid profile responses between athletic and non-athletic T2DM populations, interesting differences emerge. A systematic review on sedentary T2DM patients by Gordon et al. (33) suggest that exercise alone can produce meaningful improvements in LDL-C levels, whereas our athletic participants showed more modest changes with PA intervention alone. This discrepancy likely reflects the already-adapted lipid metabolism in recreational athletes, where additional exercise may yield diminishing returns compared to previously inactive individuals. However, our NR and PA+NR groups demonstrated substantial improvements in lipid profiles, similar to findings by Kodama et al. (31) in non-athletic populations, suggesting that dietary intervention remains crucial for lipid management regardless of physical activity status. The considerable improvement in HDL-C levels in our PA+NR group (from 36.80 to 54.20 mg/dL) generally exceeds typical responses reported in non-athletic populations by Kraus et al. (32), potentially indicating a synergistic effect between existing athletic conditioning and targeted dietary modifications that enhances HDL-C production beyond what is typically observed in sedentary individuals with T2DM.

### 4.4 Effects on insulin resistance

The PA+NR group experienced a significant decrease in HOMA-IR, an effect not achieved by the PA or NR groups alone. This finding is in line with previous studies, such as (34), which demonstrated that exercise combined with dietary modifications enhances both hepatic and peripheral insulin sensitivity in T2DM patients. The combined intervention likely reduces hepatic glucose production while increasing muscle glucose disposal, thereby providing comprehensive glycemic control.

Physical activity alone, though beneficial, has limited effects on reducing insulin resistance unless paired with dietary interventions that address excess hepatic glucose output and improve lipid profiles. For instance, Holten et al. (35) also found that insulin sensitivity improvements were significantly enhanced when exercise was paired with dietary modifications, emphasizing the necessity of a multifaceted approach to T2DM management. This suggests that clinical recommendations for T2DM should prioritize dual interventions for optimizing insulin sensitivity, as the metabolic flexibility needed to improve insulin action is best achieved through combined lifestyle changes. Insulin sensitivity improvements in our athletic population with T2DM showed both similarities and differences compared to non-athletic counterparts. Studies by Winnick et al. (34) demonstrated that combined interventions can improve insulin sensitivity in sedentary individuals with T2DM, but our PA+NR group demonstrated particularly pronounced improvements, with HOMA-IR decreasing from 6.70 to 3.20. This enhanced response may reflect the underlying metabolic flexibility developed through regular athletic training, which potentially primes insulin signaling pathways for greater adaptability when supported by appropriate dietary modifications. Conversely, our PA-only group showed more modest improvements in insulin sensitivity compared to those typically reported by Holten et al. (35) in previously inactive individuals, suggesting that recreational athletes with T2DM may have already realized some exercise-related benefits to insulin action, making additional exercise-only interventions less impactful without concurrent dietary optimization.

## 5 Strengths and practical applications

Our study investigated the effects of combined diet and physical activity on glycemic control, lipid profiles, and body composition in male recreational athletes with T2DM. The findings suggest that a combined therapeutic diet and physical activity program effectively improved body composition and biochemical parameters in recreational athletes with T2DM.

This study presents several key strengths by investigating a population that remains understudied despite the growing awareness of diabetes among physically active individuals. While a combined aerobic and resistance training program alongside dietary interventions presents a multimodal approach that has been shown to provide superior metabolic benefits compared to either intervention alone. Additionally, a 12-week duration of the intervention is sufficient to observe meaningful physiological and metabolic adaptations. Short-term interventions may not fully capture the long-term effects of combined diet and physical activity on glycemic control and body composition.

The study provides practical and evidence-based recommendations for optimizing diabetes management in active individuals. Lastly, the findings contribute to a better understanding of how structured lifestyle modifications can be tailored to athletes with T2DM, potentially influencing future clinical and sports science guidelines.

## 6 Limitations and future directions

This study has several limitations that should be acknowledged. First, the relatively small sample size may limit the generalizability of the findings to broader populations with T2DM, particularly those with varying levels of physical activity or comorbidities. Second, the study population consisted exclusively of male recreational athletes, which may limit the applicability of the results to females or non-athletic individuals with type 2 diabetes mellitus (T2DM). As a result, the findings may not be directly applicable to the broader T2DM population, including sedentary individuals or those with different physiological characteristics. Future research should include a more diverse population to enhance the external validity of the results. Third, the reliance on bioelectrical impedance for body composition assessment, while practical, may introduce measurement variability compared to more precise methods like dual-energy X-ray absorptiometry (DXA). This limitation could affect the precision of body composition changes observed in the study. Also, dietary adherence was self-reported, which may introduce reporting bias and affect the accuracy of nutritional intake data. The lack of objective dietary tracking methods, such as direct supervision or digital food tracking tools, could influence the reliability of dietary intervention outcomes.

Additionally, the absence of long-term follow-up data limits our understanding of the sustained effects of these interventions over time. Without extended monitoring beyond the 12-week intervention, it is unclear whether participants maintained their improvements in glycemic control and body composition over time. Lastly, dietary adherence and exercise intensity were self-reported, which could introduce bias. Future studies should consider controlled supervision, larger sample sizes, and more precise measurement tools to further validate these findings and assess long-term intervention effects.

## 7 Conclusions

This study demonstrated that a combined approach of physical activity and dietary modification was effective in improving body composition, glycemic control, lipid profiles, and insulin sensitivity among male recreational athletes aged 30-45 with T2DM. The findings suggested that integrating structured exercise, particularly resistance training, with individualized dietary guidance provided substantial benefits for managing glycemic levels and reducing cardiovascular risks. For clinical practice, this highlighted the practical value of lifestyle-based strategies as an essential part of diabetes care. Future research should build on these results by examining the effects across varied populations, including females and non-athletes, and evaluating the long-term impact of these interventions to enhance diabetes management recommendations.

## Data Availability

The original contributions presented in the study are included in the article/supplementary material. Further inquiries can be directed to the corresponding authors.
